# New targets for resistant prostate cancer

**DOI:** 10.18632/oncotarget.2544

**Published:** 2014-10-01

**Authors:** Timothy C. Thompson, Likun Li

**Affiliations:** Department of Genitourinary Medical Oncology, The University of Texas MD Anderson Cancer Center, Houston, Texas, USA; Department of Genitourinary Medical Oncology, The University of Texas MD Anderson Cancer Center, Houston, Texas, USA

Although prostate cancer (PCa) initially responds to standard luteinizing hormone releasing hormone agonist–based androgen-deprivation therapy (ADT), most advanced, lethal PCa eventually becomes castration-resistant PCa (CRPC) and metastasizes to bone [1]. The use of second generation anti–androgen receptor (AR) agents, i.e., abiraterone and enzalutamide (ENZ), has resulted in modestly increased survival rates, but these therapies are not curative, and novel therapeutic approaches are needed to treat this lethal disease.

The heterogeneous nature of PCa and the difficulty in obtaining sufficient clinical samples of CRPC for genomics analysis have impeded understanding of this disease. However, recent genomics studies have identified potentially actionable signaling pathways that contribute to CRPC. In addition to mutations in genes or pathways, including PI3K-Akt and Wnt/β-catenin, mutations were found in numerous proteins that interact with AR in CRPC samples [2]. These results support the longstanding concept that persistent AR signaling through mutation, amplification, and aberrant expression represents a hallmark of CRPC [1,3].

Although the fact that mutations cause persistent AR signaling has long been understood, suppression of AR signaling through pharmacologic inhibition was only recently shown to lead to derepression of specific oncogenic signaling pathways in PCa. Carver *et al* demonstrated that ADT or treatment with ENZ increased the phosphorylation of Akt in PTEN-deleted LNCaP cells and LAPC4 cells, whereas the combination of ENZ and BEZ235, a PI3K inhibitor, caused marked regression of PCa in animal models [4]. The authors demonstrated that the negative feedback signal from AR to the PI3K-Akt pathway is AR-stimulated, FKBP5-mediated activation of the Akt phosphatase PHLPP, and that this signal is interrupted by AR inhibition [4].

The concept that AR inhibition may derepress particular AR-suppressed genes was extended by the recent findings of Cai *et al*, who showed that androgen-liganded AR reduced AR target gene expression through ARBS2, a transcriptional enhancer [5]. The authors demonstrated that specific genes related to DNA metabolism, DNA synthesis and repair, androgen metabolism, and the cell cycle are repressed by active AR and derepressed by anti-AR agents [5]. Importantly, many of these derepressed genes were shown to be upregulated in castration-resistant VCaP xenografts and in CRPC bone marrow metastases from PCa patients. In addition, intratumoral synthesis of androgens was shown to lead to partial restoration of AR transcriptional activity in CRPC, yet these androgen levels were not sufficient to downregulate the expression of specific AR-repressed genes related to the cell cycle, DNA synthesis and repair, and DNA metabolism [5]. Overall, this report established that specific AR-repressed genes related to DNA metabolism, DNA synthesis and repair, and the cell cycle represent overexpressed genes in CRPC and thus potential therapeutic targets.

Recently, Arora *et al* demonstrated that glucocorticoid receptor (GR) expression was upregulated in LNCaP/AR xenografts that are resistant to ENZ, as well as in CRPC bone metastases from patients that had a relatively poor response to ENZ compared with those from patients who had a more favorable response [6]. Additional results from this study showed that GR inhibition in resistant cells can potentiate sensitivity to ENZ. The study also identified a common set of target genes regulated by AR and GR in PCa cells and showed that GR can drive AR target genes in ENZ-resistant PCa. Importantly, experimental data also indicated that AR directly represses GR expression in ENZ-resistant LNCaP/AR xenografts, suggesting a model in which ENZ-mediated GR derepression provides an adaptive resistance mechanism through which clonal expansion occurs in GR-driven CRPC [6]. These results are consistent with those of Cai *et al* [5] with regard to anti-AR–mediated derepression of genes that may ultimately drive therapy resistance.

Li *et al* recently found that ENZ increased expression of the proto-oncogene *c-MYB*, a transcription factor that has been shown to be upregulated in multiple malignancies [7]. The results of this study showed that c-Myb increases growth and metastatic potential of both AR-positive and AR-negative PCa cells [7]. c-Myb expression was increased in AR-positive, androgen-independent PCa cells compared with PCa cells that were dependent on androgens for growth. Importantly, the results of this study showed that AR and c-Myb share a common set of target genes that include a DNA damage response signature that is strongly associated with cancer recurrence, castration resistance, and metastatic disease [7]. These results point to a mechanistic model in which ENZ-mediated derepression of c-Myb expression compensates for loss of AR activity by ENZ treatment through regulation of their common DNA damage response gene targets, which, in turn, mediates resistance to ENZ. Further experiments revealed that c-Myb regulates DNA damage response through Topbp1, the ataxia-telangiectasia and Rad3–related protein (ATR), and Chk1 protein, which regulate DNA damage response checkpoints [7]. On the basis of these results, the authors devised a combination therapeutic strategy with an inhibitor of c-Myb-Topbp1-ATR-Chk1 signaling, i.e., the Chk1 inhibitor AZD7762, and an inhibitor of poly(ADP-ribose) polymerase (PARP), i.e., olaparib. Results of in vitro and in vivo studies showed that this combination has synergistic effects in PCa models [7]. Thus, this study identified a gene signature which is co-regulated by AR and c-Myb, and which specifies a therapeutically actionable pathway for CRPC.

**Figure 1:**
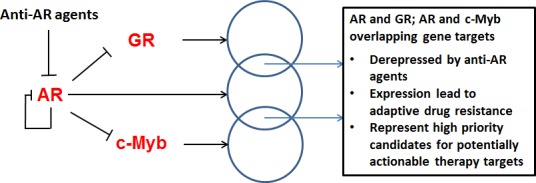
Overlapping androgen receptor (AR) and glucocorticoid receptor (GR) or overlapping AR and c-Myb target genes as therapy targets for castration-resistant prostate cancer Recent studies point to a high priority gene set of potentially actionable therapy targets for castration-resistant prostate cancer. Anti-AR agents result in derepression of specific genes, including targets of GR and c-Myb, that function as transcriptional regulators of a subset of target genes that overlap with target genes of AR. Expression of these genes is related to adaptive drug resistance.

Overall, the results of these recent studies (i) demonstrate the persistence and importance of AR-regulated gene expression in CRPC; (ii) support a model of anti-AR agent–mediated gene derepression as an adaptive resistance mechanism; (iii) suggest that this adaptive response is linked to further progression of a therapy-resistant cell population; and (iv) suggest that overlapping AR and GR, and AR and c-Myb, target genes are strongly selected for drug resistance and should be considered for further study as potential therapy targets for CRPC (Figure 1).
